# Anti-*Legionella dumoffii* Activity of *Galleria mellonella* Defensin and Apolipophorin III

**DOI:** 10.3390/ijms131217048

**Published:** 2012-12-12

**Authors:** Marta Palusińska-Szysz, Agnieszka Zdybicka-Barabas, Bożena Pawlikowska-Pawlęga, Pawel Mak, Małgorzata Cytryńska

**Affiliations:** 1 Department of Genetics and Microbiology, Institute of Microbiology and Biotechnology, Maria Curie-Sklodowska University, Akademicka 19 St., 20-033 Lublin, Poland; 2 Department of Immunobiology, Institute of Biology and Biochemistry, Maria Curie-Sklodowska University, Akademicka 19 St., 20-033 Lublin, Poland; E-Mails: barabas@poczta.umcs.lublin.pl (A.Z.-B.); cytryna@poczta.umcs.lublin.pl (M.C.); 3 Department of Comparative Anatomy and Anthropology, Institute of Biology and Biochemistry, Maria Curie-Sklodowska University, Akademicka 19 St., 20-033 Lublin, Poland; E-Mail: bozka1996@o2.pl; 4 Department of Analytical Biochemistry, Faculty of Biochemistry, Biophysics and Biotechnology, Jagiellonian University, Gronostajowa 7 St., 30-387 Krakow, Poland; E-Mail: pawel.mak@uj.edu.pl

**Keywords:** phosphatidylcholine, *Legionella*, *Galleria mellonella*, apolipophorin III, *Galleria* defensin

## Abstract

The gram-negative bacterium *Legionella dumoffii* is, beside *Legionella pneumophila*, an etiological agent of Legionnaires’ disease, an atypical form of pneumonia. The aim of this study was to determine the antimicrobial activity of *Galleria mellonella* defense polypeptides against *L. dumoffii*. The extract of immune hemolymph, containing a mixture of defense peptides and proteins, exhibited a dose-dependent bactericidal effect on *L. dumoffii*. The bacterium appeared sensitive to a main component of the hemolymph extract, apolipophorin III, as well as to a defense peptide, *Galleria* defensin, used at the concentrations 0.4 mg/mL and 40 μg/mL, respectively. *L. dumoffii* cells cultured in the presence of choline were more susceptible to both defense factors analyzed. A transmission electron microscopy study of bacterial cells demonstrated that *Galleria* defensin and apolipophorin III induced irreversible cell wall damage and strong intracellular alterations, *i.e*., increased vacuolization, cytoplasm condensation and the appearance of electron-white spaces in electron micrographs. Our findings suggest that insects, such as *G. mellonella*, with their great diversity of antimicrobial factors, can serve as a rich source of compounds for the testing of *Legionella* susceptibility to defense-related peptides and proteins.

## 1. Introduction

Legionellae are fastidious gram-negative bacteria found in moist natural environments as intracellular parasites of freshwater protozoa [1]. The bacteria also occur in man-made aquatic systems, which facilitate proliferation and dissemination of these bacteria by producing water-air aerosol. After transmission to humans, *Legionella* spp. can cause a pneumonia called Legionnaires’ disease. The majority of cases of Legionnaires’ disease are caused by *Legionella pneumophila*, but *Legionella dumoffii* is the fourth most common causative agent [2]. In humans, *L. dumoffii* causes even more serious and rapidly progressive types of pneumonia than that induced by other strains of *Legionella*[3]. Moreover, infection by *L. dumoffii* can contribute to other diseases, such as septic arthritis, pericarditis and prosthetic-valve endocarditis [4,5]. The ability of *L. dumoffii* to survive and multiply within protozoa [6,7] and in a human macrophage cell line has been described [8]. There are also suggestions that *L. dumoffii* can invade and proliferate in human alveolar epithelial cells [9]. To date, the mechanisms underlying the virulence and rapid progression of pneumonia due to *L. dumoffii* are poorly understood.

As an intracellular pathogen, *Legionella* spp. has evolved multiple strategies needed to overcome or evade the defense system of the host cell. For example, after being phagocytosed by mammalian macrophages or amoebae, *L. pneumophila* resides in a phagosome, whose ability to fuse with lysosomes is virtually restricted. One of the most important compounds of the host defense system produced to kill the invading pathogens are cationic antimicrobial peptides. Human β-defensins (hBDs), the members of the β-defensin family that display antimicrobial and immunomodulatory properties, are probably the essential defense factors of epithelial mucosa and macrophages [10]. It was demonstrated that although extracellular *L. pneumophila* was resistant to the cationic peptide antibiotic—polymyxin B—it was sensitive to hBD-2 and hBD-3 defensins, suggesting that these peptides are directly involved in the defense against *Legionella* in humans [10,11].

From among one thousand eukaryotic antimicrobial peptides characterized to date, about 200 have been described in insects. Insects have developed a very effective innate immunity system in which invading pathogens are efficiently eliminated by antimicrobial peptides. There are three main classes of cationic defense peptides in insects: (i) α-helical peptides without cysteine residues, e.g., cecropins, (ii) peptides with a structure stabilized by disulfide bridges, e.g., defensins, and (iii) peptides with overrepresentation of one amino acid, e.g., apidaecins or drosocins [12]. The structure of most of the insect defensins, similarly to their human counterparts, is stabilized by three disulfide bridges formed by six cysteine residues. However, in contrast to hBDs containing an αβββ scaffold, the insect defensins contain an αββ scaffold consisting of an α-helical domain and two anti-parallel β-strands in which the α-helix is stabilized by two disulfide bridges to one strand of the β-sheet. The insect defensins are active against various bacteria, yeasts and filamentous fungi; however, their activity towards *Legionella* has not been investigated [13]. Interestingly, Casteels *et al*. [14] presented evidence that insect proline-rich peptides, apidaecins, inhibited growth of *L. pneumophila*, indicating that other defense peptides, in addition to defensins, could be involved in overcoming *Legionella* infections.

In hemolymph of the greater wax moth *Galleria mellonella* larvae, an impressive set of cationic defense peptides differing in biochemical and antimicrobial properties was reported: insect defensins, cecropins, moricins, gloverins and proline-rich peptides. In addition to many cationic ones, two anionic defense peptides were characterized in this insect [15–21]. Among eight peptides isolated by us from larval hemolymph and tested against selected gram-negative bacteria (*Escherichia coli* D31, *E. coli* ATCC 25922, *Salmonella* Typhimurium), only cecropin d-like peptide inhibited growth of *E. coli* D31 [17]. Apart from antimicrobial peptides, certain hemolymph proteins, such as hydrophobic apolipophorin III (apoLp-III), an insect homologue of human apolipoprotein E (apoE), also exhibited antibacterial activity. Both proteins apoLp-III and apoE, in addition to functioning in lipid transport, play an important role in immunity. It was reported that *G. mellonella* apoLp-III inhibited growth of gram-negative bacteria *S.* Typhimurium and *Klebsiella pneumoniae*[22–24], whereas apoE was demonstrated to be involved in immune response against *K. pneumoniae* and *Listeria monocytogenes*, as well as protection against a lethal dose of *Salmonella minnesota* LPS [25–29].

It has been documented that the antimicrobial properties of cationic defense peptides are dependent on their positive charge and amphipathicity, which enable interactions with negatively charged membrane phospholipids [30,31]. Our unpublished results demonstrated that cultivation of *L. dumoffii* on a medium supplemented with choline led to an increase in the phosphatidylcholine (PC) content in the bacterial cell membrane. In the present study, we demonstrate evidence of anti-*L. dumoffii* activity of two antimicrobial components of *G. mellonella* hemolymph, apoLp-III and *Galleria* defensin. In addition, to provide more insight into the mechanism of interaction of *G. mellonella* polypeptides with cells of *L. dumoffii*, the effect of an increased PC content in the cell membrane on the effectiveness of their antibacterial action was investigated. The effects of the action of polypeptides on bacterial cells were imaged by transmission electron microscopy. The results presented suggest that the selected defense peptides and proteins of *G. mellonella* could be used as a template for designing novel anti-*L. dumoffii* agents.

## 2. Results

### 2.1. The Effect of Extracts of Immune *G. mellonella* Hemolymph on *L. dumoffii* Survival Rate

**Figure 1:**
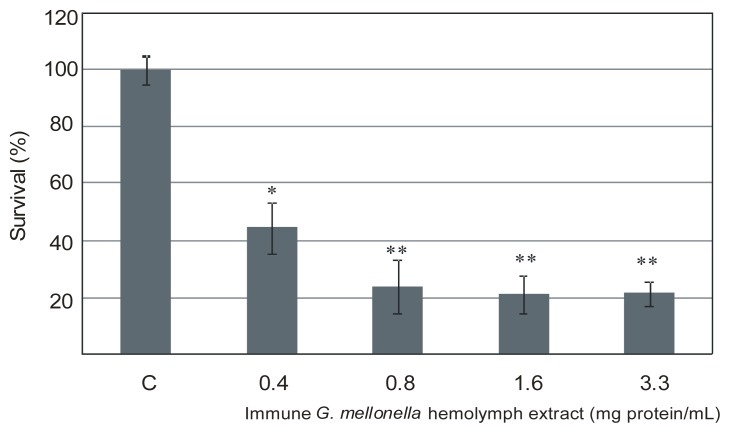
Growth inhibition of *L. dumoffii* by *G. mellonella* immune hemolymph extract. The bacteria were incubated with the extract at the concentrations 0.4–3.3 mg/mL (total protein) for 1 h, as described in the Experimental Section. Next, the cells were seeded on the agar plates, and the growing colonies were counted. Survival of the untreated cells was regarded as 100% (C; control). Statistical significance: * *p* < 0.05; ** *p* < 0.01.

Our previous study reported that methanolic extracts of *G. mellonella* immune hemolymph contain an abundant hemolymph protein, apoLp-III, as a main component, and a set of defense peptides differing in biochemical and antimicrobial properties [17,32]. A preliminary study performed using a radial diffusion assay revealed inhibition of *L. dumoffii* growth by the *G. mellonella* immune hemolymph extract as a clear zone around the point of extract application (data not shown). Then, the inhibitory effect of the extract on *L. dumoffii* cells was examined using a colony-counting assay (Figure 1). The extract reduced the survival rate of the bacteria in a dose-dependent manner in the total protein concentration range of 0.4–0.8 mg/mL. The bacteria survivability was decreased by more than 50% at the concentration 0.4 mg/mL of the total extract protein. With the increasing concentration of the protein, the bactericidal activity of the extract was enhanced, as only 25% of the bacteria survived at the protein concentration of 0.8 mg/mL (Figure 1). However, further increasing the protein concentration to 1.6 mg/mL reduced the bacteria survival only by another 5%, whereas 3.3 mg/mL did not cause a further decrease in the bacteria survival rate, probably due to protein aggregation. The data presented were obtained after 1 h incubation of the bacteria in the presence of the hemolymph extract; however, the same bactericidal effect was observed already after 15 min of incubation of the bacteria with the extract (data not shown).

### 2.2. The Effect of *G. mellonella* Defensin and apoLp-III on Survival Rate of *L. dumoffii* Cultured with and without Choline Supplementation

**Figure 2:**
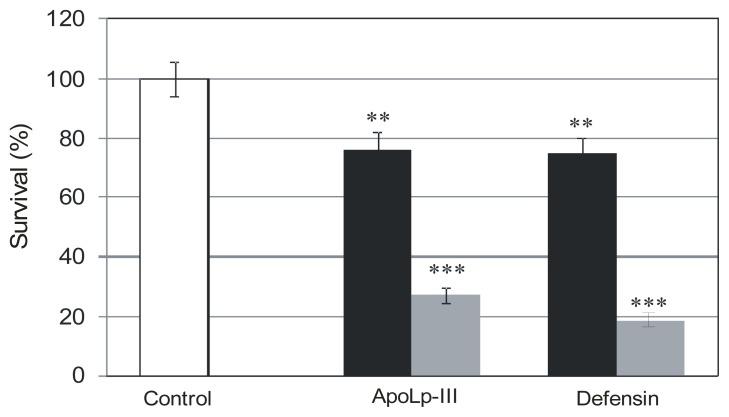
The effects of *G. mellonella* apoLp-III and defensin on *L. dumoffii* survival and influence of choline supplementation on the activity of the antimicrobial factors. The bacteria cultured on the non-supplemented (black bars) and choline-supplemented (grey bars) medium were exposed to *Galleria* defensin (40 μg/mL) or apoLp-III (0.4 mg/mL), as described in the Experimental Section. After seeding of the bacteria on the agar plates, the growing colonies were counted. Survival of the untreated cells was regarded as 100% (C; control). The results are given as mean ± SD from three independent experiments. Statistical significance: ** *p* < 0.01; *** *p* < 0.001.

The activity of *G. mellonella* defensin and apoLp-III was tested against bacteria cultured on the non-supplemented, as well as choline-enriched, BCYE medium using a colony counting assay. The bacteria were incubated for 1 h in the presence of *Galleria* defensin in the final concentration range of 4–40 μg/mL. The effect of apoLp-III, the main component of hemolymph extract, was also evaluated at the concentration of 0.4 mg/mL. The sensitivity of *L. dumoffii* to both *G. mellonella* antimicrobial factors is presented in Figure 2. The bacteria appeared moderately sensitive to *Galleria* defensin (40 μg/mL), as well as apoLp-III (0.4 mg/mL). Each of the factors decreased the survival rate of *L. dumoffii* by *ca*. 30%. For concentrations below the maximal tested, *Galleria* defensin and apoLp-III were not effective in killing *L. dumoffii* (data not shown). As apoLp-III constitutes the main component of the hemolymph extract, these results could explain the need for using relatively high concentrations of the total extract protein in our experiments presented in Figure 1. Interestingly, increased sensitivity to both defense factors was observed when *L. dumoffii* cells were cultured in the presence of choline. As shown in Figure 2, *Galleria* defensin and apoLp-III were 3.8-fold and three-fold more active, respectively, against *L. dumoffii* in respect to bacteria grown on the non-supplemented medium.

### 2.3. Transmission Electron Microscope Observations of *Legionella dumoffii*

#### 2.3.1. The Changes in the *L. dumoffii* Cell Morphology under the Influence of *G. mellonella* Immune Hemolymph Extract

**Figure 3:**
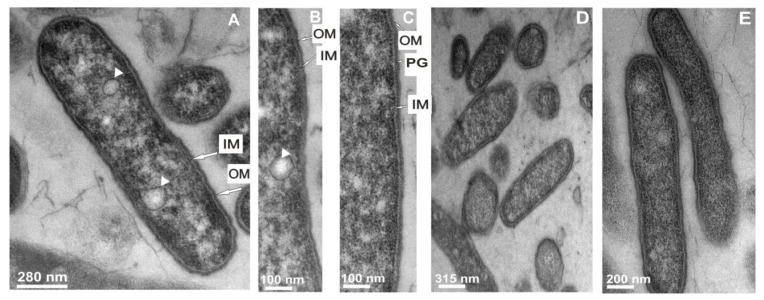

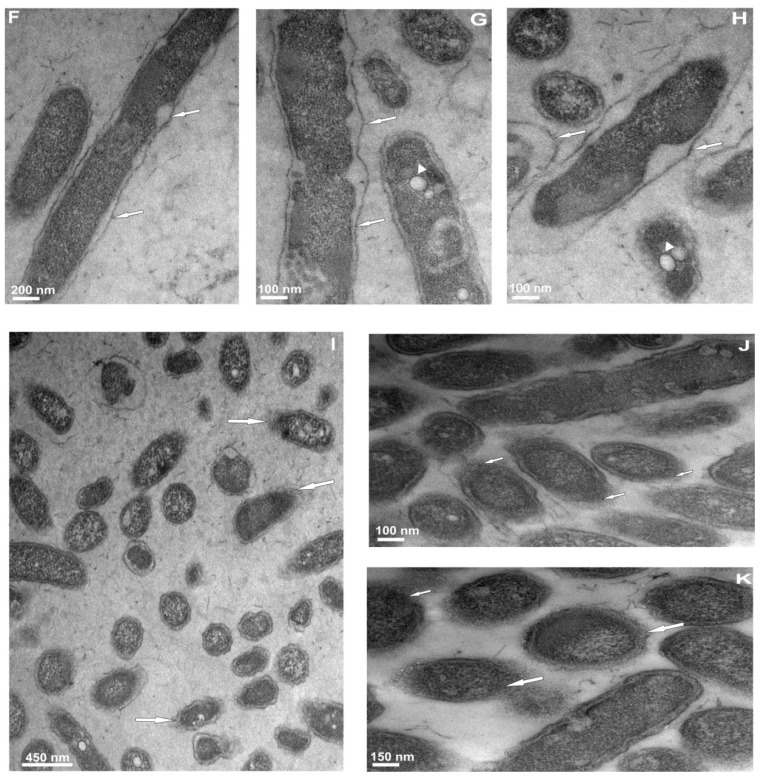
The influence of the extract of *G. mellonella* immune hemolymph on *L. dumoffii* cell morphology. The cells growing on the non-supplemented (**A**–**C**,**F**–**I**) and choline-supplemented (**D**,**E**,**J**–**K**) agar medium were exposed to *G. mellonella* hemolymph extract (**F**–**I**,**J**,**K**) or left untreated (**A**–**C**,**D**,**E**). Then, the cells were prepared for TEM analysis as described in the Experimental Section. (**A**) one big bacterium in longitudinal section; vacuoles are visible inside the cell (arrowheads); the outer and inner membrane is distinguishable in the cell envelope; (**B**) a fragment of the bacterium with a visible internal membrane (arrows); IM, inner membrane; OM, outer membrane; (**C**) a portion of the bacterium with a peptidoglycan-like layer (denoted as PG); outer and inner membranes are seen; (**D**,**E**) cells cultured on the choline-supplemented medium with a typical appearance; (**F**) cells showing cell wall damage and a periplasmatic space (arrows); (**G**) bacteria with cell wall damage (arrows) and dense cytoplasm with vacuoles (arrowhead); (**H**) enlarged view of a bacterium with strong cell envelope damage and cytoplasm condensation; the rest of the cells exhibit cell wall damage and presence of vacuoles (arrowhead); (**I**) many bacterial cells demonstrating loose attachment of the cell membrane (arrows); and (**J**,**K**) cells cultured on choline exposed to the extract with visible deterioration of the cell wall.

To demonstrate the direct effect of the extract of *G. mellonella* hemolymph on *L. dumoffii* cell morphology, the bacterial cells were observed by transmission electron microscopy. Microscopic observations of control cells (non-treated with the extract) revealed presence of small vacuoles, well discernible outer and inner membrane and cytoplasm containing numerous ribosomes. The cells were of longitudinal shape. The bacilli were usually 1.5 to 2.0 μm in length and 0.4 to 0.5 μm in diameter, and no infolding of the cytoplasmic membrane (“mesosomes”) was seen (Figure 3A–C). No changes in the structure were demonstrated in cell morphology of bacteria cultured on the choline-supplemented medium. All their organelles still had a typical appearance (Figure 3D,E). Microscopic examination of the bacteria treated with the extract of *G. mellonella* hemolymph revealed the presence of severe cell envelope damage with apparent local widening and changes in the cytoplasm appearance (Figure 3F–I). The cytoplasm was often granular and denser in comparison to the cytoplasm of the control cells. An enlarged periplasmatic space accompanying a locally widened envelope membrane was noted (Figure 3F,H). After the exposure of *L. dumoffii* to the *G. mellonella* extract, the shape of the bacteria was also modified. Additionally, loose attachment of the cell membrane was visible (arrows). The vacuoles were electron-lucent and surrounded by an electron-dense membrane (arrowheads) (Figure 3G,H). Similar changes, especially in the cell wall, were observed in bacterial cells cultured on the choline-supplemented medium after exposure to the *G. mellonella* extract. Distortion and loss of the cell wall in some parts of the cells were noted (Figure 3J,K).

#### 2.3.2. Ultrastructural Changes in the Cells of *L. dumoffii* after Treatment with Defensin and apoLp-III Isolated from *G. mellonella* Hemolymph

**Figure 4:**
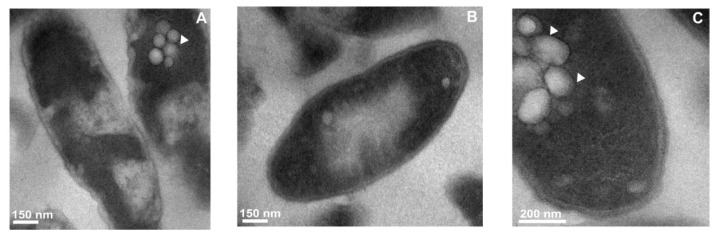

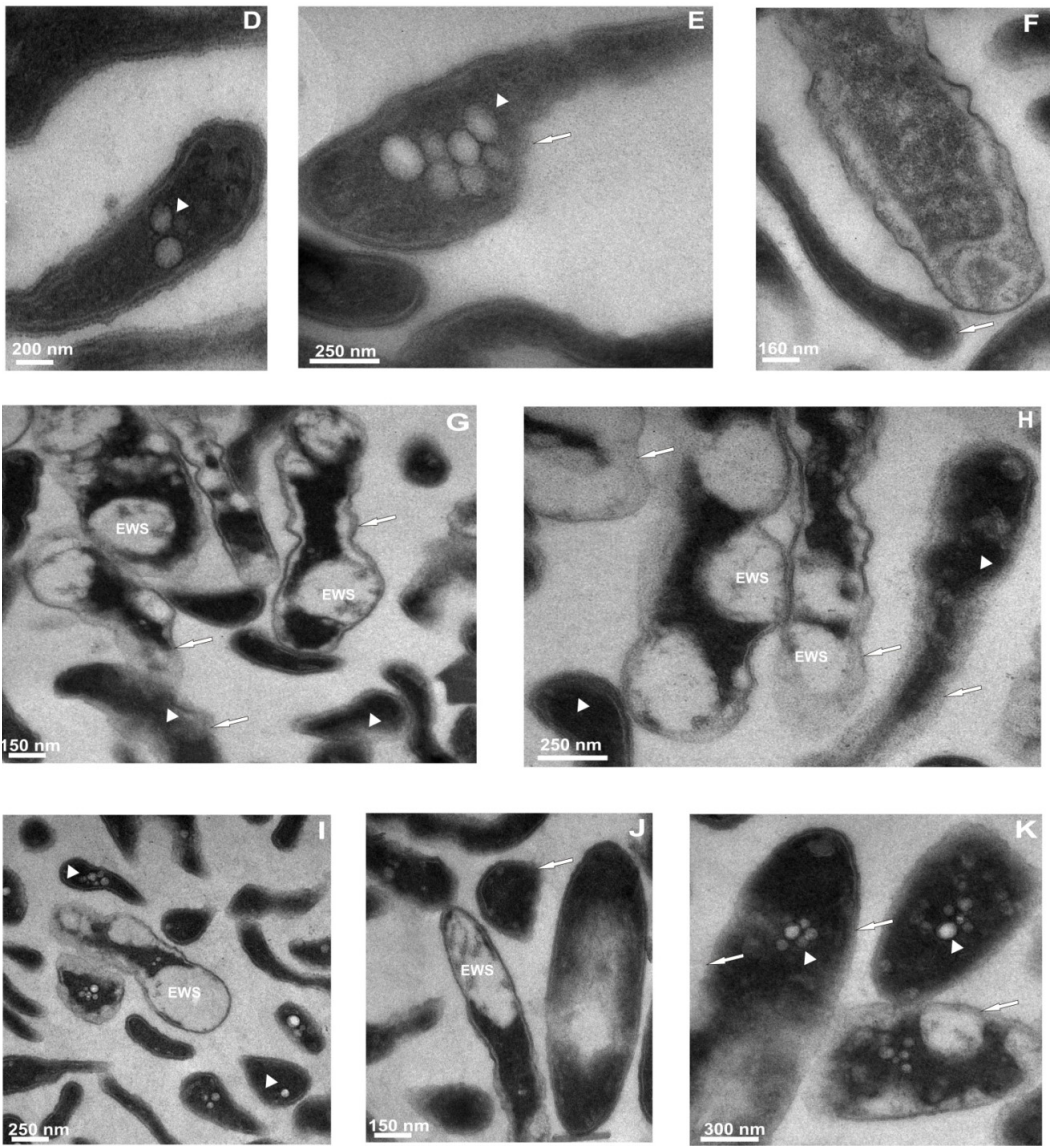
The influence of defensin and apolipophorin III isolated from *G. mellonella* hemolymph on the ultrastructure of *L. dumoffii* cells. The cells grown on the non-supplemented (**A**–**C**,**G**,**H**) and choline-supplemented (**D**–**F**,**I**–**K**) medium were incubated in the presence of apoLp-III (**A**–**F**) or *Galleria* defensin (**G**–**K**). Then the cells were prepared for TEM analysis as described in the Experimental Section. (**A**,**B**) cells showing condensed cytoplasm, regions with decreased electron density and the presence of vacuoles (arrowhead); (**C**) a fragment of the bacterium with a group of visible vacuoles and dark, dense cytoplasm; (**D**,**E**) cells with vacuolization features (arrowheads) and cell envelope damage (arrow); (**F**) bacteria with cell wall distortion (arrow) and a widened periplasmatic space; (**G**) cells with electron-white spaces (EWS), membrane deterioration (arrows), and condensed content (arrowheads); (**H**) irreversible cell wall damage visible in bacteria (arrows) together with dense areas of cytoplasm or entire cytoplasm of the whole cells (arrowheads); (**I**) bacteria demonstrating loss of cell wall integrity, vacuolization of cytoplasm (arrowheads), cell shrinkage; and (**J**,**K**) cells showing cell wall damage (arrows), electron-white spaces (EWS), presence of small vacuoles (arrowheads).

In the subsequent stage of the microscopic examination, the bacteria cultured on the non-supplemented and choline-enriched medium were treated with purified *Galleria* defensin or apoLp-III. The microscopic observations revealed significant changes in the ultrastructural characteristics of *L. dumoffii* exposed to apoLp-III, such as the presence of many vacuoles within the cytoplasm and minor changes in the cell envelope structure (Figure 4). In various parts of the cell, the cytoplasm was condensed and, additionally, regions with decreased electron density were detected (Figure 4A–C). The cells cultured on the choline-supplemented medium also revealed vacuolization of the cytoplasm after the treatment with apoLp-III. However, the cell envelope damage was greater than in the cells exposed to apoLp-III, but cultured on the medium without choline supplementation. Some cells exhibited a widened periplasmatic space (Figure 4D–F). Distinct changes were observed in the ultrastructure of cells treated with *Galleria* defensin and apoLp-III. Defensin treatment of *L. dumoffii* cells considerably affected the cell morphology of *L. dumoffii*, and the loss of typical subcellular organization was detected. Within the cytoplasm, large electron-white spaces (vacuole-like spaces), as well as highly condensed areas, were observed. Substantial, irreversible (lethal) cell wall damage was noticed. Some cells were shrunk, with a very dense content and barely discernible cell envelope (Figure 4G,H). The cells of bacteria cultured on the choline-enriched medium showed substantial changes due to *Galleria* defensin treatment. Cell shrinkage, condensation of cytoplasm and loss of cell envelope integrity were followed by the appearance of small vacuoles surrounded by an electron-dense membrane in numerous shrunken cells (Figure 4I). In cells larger in size, further destruction occurred, such as deprivation of the cell wall structure and the appearance of electron-white regions. Additionally, small vacuoles were visible in the dense cytoplasm (Figure 4J,K).

## 3. Discussion

In the present study, the effects of *G. mellonella* defense peptides and proteins, essential components of insect immune response, on the viability of *L. dumoffii* were investigated. To the best of our knowledge, this is the first report on the antibacterial activity of insect defense factors against this species of *Legionella. L. dumoffii* was found to be sensitive to *Galleria* defensin. The inhibitory effect of *Galleria* defensin on *L. dumoffii* is the first evidence of the antibacterial activity of this peptide. In previous studies, only the antifungal activity of *Galleria* defensin was reported [15,17]. Recently, *G. mellonella* larvae have been exploited as a model system to study *L. pneumophila* pathogenicity, however, susceptibility of the bacteria to *G. mellonella* antimicrobial proteins and peptides has not been tested [33]. Interestingly, infection of the larvae by *L. pneumophila* upregulated expression of *Galleria* defensin, suggesting involvement of this peptide in anti-*Legionella* response, which is consistent with our results demonstrating its anti-*Legionella* activity *in vitro*.

Antibacterial activity of *G. mellonella* apoLp-III against selected bacteria (*Bacillus circulans*, *S.* Typhimurium, *K. pneumoniae*) was reported in our previous papers [23,24]. Inhibition of *L. dumoffii* growth by apoLp-III is another piece of evidence for the antibacterial activity of this insect protein. Moreover, susceptibility of *L. dumoffii* to apoLp-III could suggest involvement of its human homologue, apoE, in anti-*Legionella* defense in humans.

Like other *Legionella* species (*L. pneumophila*, *L. bozemanae*, *L. lytica*), *L. dumoffii* utilizes extracellular choline for the synthesis of phosphatidylcholine (PC)—an important constituent of the cell envelope [34,35]. PC is primarily a component of membrane lipids in eukaryotic cells; only 15% of bacteria, mainly photosynthetic bacteria with an extensive internal membrane structure or the host cell-associated bacteria (either pathogenic or symbiotic), contain this phospholipid in their cell envelope. Aside from the role in membrane assembly, PC functions also as a modulator of a wide variety of cellular pathways. Exposure of phosphocholine groups, characteristic for bacteria residing predominantly in the respiratory tract, allows the bacteria to interact with the platelet-activating factor (PAF) receptor of the host and, consequently, to invade the epithelial cells [36].

In our experiments, the supplementation of *L. dumoffii* culture with choline led to the significantly increased susceptibility of the bacteria to apoLp-III and *Galleria* defensin. The results indicated the relationship between the increased content of the phospholipid in the bacterial cell membrane and the effectiveness of antimicrobial activity of the *G. mellonella* defense compounds tested. One could suggest enhanced binding of both defense factors to the cells of *L. dumoffii* grown in the presence of choline. Due to the fact that PC represents zwitterionic phospholipids, it seems that the interaction of *Galleria* defensin, as well as apoLp-III with *L. dumoffii* cells, was enhanced mainly by the increased hydrophobic properties of the bacterial cell envelope. Moreover, one could postulate PC to be a receptor for binding these *G. mellonella* defense factors to the *L. dumoffii* cell membrane. For example, it was reported that molecules of the synthetic antimicrobial peptide cecropin B3 containing two hydrophobic α-helical segments, clustered in the domains of the membrane enriched in lipids with neutral headgroups, such as PC [37]. It was also suggested that phosphorylcholine decorations may change the sensitivity of *Haemophilus influenzae* bacteria to the antimicrobial activity of the human peptide LL-37/hCAP18 [38]. Recently, it has been reported that the increase of branched-chain fatty acids and the decrease of fatty acid chain length in the *L. pneumophila* membrane were correlated with the increased resistance of the bacteria to warnericin RK, an amphiphilic α-helical antimicrobial peptide produced by *Staphylococcus werneri*[39]. Hence, sensitivity of the bacteria to antimicrobial peptides and proteins would be, at least in part, modulated by the membrane fatty acid composition.

In gram-negative bacteria, such as *S.* Typhimurium and *Pseudomonas aeruginosa*, antimicrobial peptides comprise environmental signals sensed by two-component systems, PhoPQ, PmrAB and ParRS, respectively. Activation of these systems is responsible for the regulation of gene expression, leading to LPS modification and adaptive resistance to cationic antimicrobials [40–42]. In *Legionella*, regulation of LPS modification genes by two-component systems has not been yet reported, however a role for the PmrAB system in the regulation of several genes encoding Dot/Icm-secreted effectors has been shown recently in *L. pneumophila*[43]. In addition, the involvement of the *rcp* gene in *L. pneumophila* resistance to antimicrobial peptides and polymyxin B has been demonstrated [44]. Rcp protein exhibits strong sequence and functional homology to *S.* Typhimurium PagP protein, acting as a palmitoyl transferase able to modify the lipid A component of LPS by the addition of fatty acid palmitate. Such modification is believed to promote resistance to cationic antimicrobial peptides by decreasing membrane fluidity and preventing insertion of the peptides. It is possible that Rcp protein plays a similar role in *L. pneumophila*[44]. Taking this into consideration, the lower susceptibility of *L. dumoffii* cells cultured without choline supplementation to *G. mellonella* defense factors reported in our study could imply sensing of the antimicrobial peptides by *L. dumoffii* cells and induction of expression of genes involved in the bacteria response to these molecules. Explanation of the impact of antimicrobial peptides on transcriptional regulation of the virulence factors in *Legionella* requires further detailed investigations. The destructive effect of the extract of *G. mellonella* hemolymph, apoLp-III and defensin on *L. dumoffii* cell morphology was observed by transmission electron microscopy. The differences between the ultrastructural changes induced by apoLp-III and by *Galleria* defensin are possibly related to the diverse mechanisms of action of these polypeptides against *L. dumoffii*. Further experimental investigations are needed to explain the mode of action of apoLp-III and *Galleria* defensin on the cells of *L. dumoffii* and the role of PC in bacterial sensitivity to these compounds.

## 4. Experimental Section

### 4.1. Bacterial Strain and Culture Conditions

*L. dumoffii*, strain ATCC 33279 was kindly supplied by Dr. B. Fields and Dr. E. Brown from CDC Atlanta (GA, USA). The bacteria were cultured for three days at 37 °C in humid atmosphere and 5% CO_2_ on buffered charcoal yeast extract (BCYE) agar supplemented with the Growth Supplement SR110 (Oxoid, Basingstoke, Hampshire, UK) [45]. For growth of bacteria on the choline-enriched medium, BCYE agar was supplied with 100 μg/mL choline chloride (Sigma-Aldrich, Chemical Co., St. Louis, MO, USA). The bacteria collected from this medium were washed three times with water by intensive vortexing and centrifugation at 8000× *g* for 10 min.

### 4.2. Insect Culture and Immune Challenge

Larvae of the greater wax moth *G. mellonella* (Lepidoptera: Pyralidae) were reared on a natural diet—honeybee nest debris at 30 °C in the dark. Last instar larvae (250–300 mg in weight) were used throughout the study. For immune challenge, the larvae were pierced with a needle dipped into a pellet of viable *E. coli* D31 and *Micrococcus luteus* ATCC 10240 cells. The larvae were kept at 30 °C in the dark, and the hemolymph was collected 24 h after the treatment.

### 4.3. Preparation of Methanolic Extracts of *G. mellonella* Hemolymph

Acidic/methanolic extracts were prepared from the hemolymph of immune-challenged *G. mellonella* larvae. The cell-free hemolymph was diluted ten times with an extraction solution consisting of methanol:glacial acetic acid:water (90:1:9 *v*/*v*/*v*). Precipitated proteins were pelleted by centrifugation at 20,000× *g* for 30 min at 4 °C. The supernatant obtained was collected and freeze-dried, and the resulting lyophilisate was dissolved in 0.1% trifluoroacetic acid (TFA). For removal of lipids from the extract, the same volume of n-hexane was added, and the sample was vortexed and centrifuged at 20,000× *g* for 10 min at 4 °C. The upper fraction containing lipids was removed, and an equal volume of ethyl acetate was added. After vortexing and centrifugation, the water fraction was freeze-dried and stored at −20 °C until use. For the experiments, the extracts were dissolved in apyrogenic water (2/3 volume of hemolymph). The hemolymph extracts contained proteins with a molecular weight below 30 kDa (mainly apoLp-III) and defense peptides, as demonstrated in our previous paper [17].

### 4.4. Purification of *G. mellonella* Defense Peptides and Proteins

The defense peptides and proteins were purified from the methanolic extracts of the immune-challenged *G. mellonella* larvae hemolymph according to the modified methods described previously by Cytryńska *et al*. [17]. The lyophilized and deprived of lipids immune hemolymph extract was reconstituted in 0.1% (*v*/*v*) trifluoroacetic acid (TFA) and subjected to the first step of purification using a Discovery Bio Wide Pore C18 250 mm × 4.6 mm column (Sigma) and a two buffer set: A: 0.1% TFA (*v*/*v*), B: 0.07% TFA, 80% acetonitrile (*v*/*v*). The linear 30%–70% gradient of buffer B over 35 min, 1 mL/min flow rate, and spectrophotometric detection at 220/280 nm was applied. This and all the following chromatographic steps were performed on a P680 HPLC system (Dionex, Munchen, Germany). Six consecutive fractions were collected: anionic peptide 1, lysozyme, a mixture of proline rich peptide 2 and apolipophoricin, *Galleria* defensin, a mixture of anionic peptide 2 and cecropin d-like peptide, as well as apolipophorin III. The fraction containing apolipophorin III was homogenous and was used without further purification. The fraction containing *Galleria* defensin was rechromatographed using the same column as above and the 39%–42% of buffer B over 25 min. The purity and identity of the collected fractions were confirmed by SDS-PAGE electrophoresis and *N*-terminal sequencing. In brief, the electrophoresis was performed according to the method of Schägger and von Jagow [46], the gel obtained was electroblotted onto a polyvinylidene difluoride (PVDF) membrane (Immobilon PSQ, Millipore, Billerica, MA, USA) stained with Coomassie Brillant Blue R-250, and then the peptides were identified by Edman degradation on a Procise 491 automatic protein sequencer (Applied Biosystems, Foster City, CA, USA). Purified *Galleria* defensin and apoLp-III were freeze-dried and stored at −20 °C until use. Before antimicrobial activity tests, they were dissolved in apyrogenic water.

### 4.5. Anti-Legionella Activity Assays

#### 4.5.1. Radial Diffusion Assay

Ten MicroLitre (μL) of bacterial suspension adjusted to an optical density at 600 nm (OD_620_) of 0.1 was placed on a BCYE agar and 5 μL of the *G. mellonella* hemolymph extract containing 0.125 mg of total protein, were applied centrally. After a three-day incubation of the plates at 37 °C, the bacterial growth inhibition zone, as a clear zone around the point of the extract application, was evaluated.

#### 4.5.2. Colony Counting Assay

Ten-fold serial dilutions were made till a dilution of 10^−4^ from the bacterial suspension of an optical density OD_620_ of 0.1 corresponding to 2 × 10^8^ cells/mL. Next, 5μL of the last dilution was transferred into sterile Eppendorf tubes and the *G. mellonella* hemolymph extract, purified apoLp-III or *Galleria* defensin to the final concentration in the range 0.4–3.3 mg/mL, 0.4 mg/mL and 4–40 μg/mL, respectively, were added. The bacteria were preincubated with the hemolymph extract or the polypeptides for 1 h at 37 °C, and then placed on the BCYE plates. After a three-day incubation at 37 °C, the grown colonies were counted. The experiments with bacteria cultured on choline-enriched medium were performed in the same way. All the experiments were conducted in three replicates. The results were referred to the controls defined as the total (100%) survival of *L. dumoffii* cells incubated in water without the addition of the hemolymph extract or polypeptides under the same conditions.

### 4.6. Preparation of Samples for Microscopic Analysis

The bacteria growing at the periphery of the clear growth inhibition zones were used for microscope observations of the effects exerted on *L. dumoffii* cell morphology by the *G. mellonella* hemolymph extract. As the control, the bacterial cells, well growing in a form of turf at a distance of 2 cm from the edge of the growth inhibition zone, were collected from the same agar plate. In order to check the influence of particular peptides, *Galleria* defensin or apoLp-III at the final concentration of 40 μg/mL or 0.4 mg/mL, respectively, were added to four tubes containing 50 μL of suspension of bacteria cultured on BCYE of an OD_620_ of 0.1 and to four tubes containing bacteria that were cultured on choline-supplemented BCYE of the same optical density. After a 1 h incubation at 37 °C, the suspensions incubated in the presence of the particular factors were pooled and centrifuged at 8000× *g* for 10 min. The resulting bacterial pellets were used for microscopic analyses.

### 4.7. Transmission Electron Microscopy Analysis of *L. dumoffii* Cells

The bacterial cells exposed to *G. mellonella* hemolymph extract were gently scraped from the agar surface using the microbiological loop and collected into the vials containing 2% formaldehyde (freshly prepared from paraformaldehyde) and 2.5% glutaraldehyde dissolved in 0.1 M sodium cacodylate buffer (pH 7.4). Then, the cell suspension was centrifuged at 8000× *g* for 10 min. The cell pellets were fixed for 24 h at 4 °C in buffered glutaraldehyde and formaldehyde (2.5% and 2%, respectively). After rinsing several times with 0.1 M cacodylate buffer (pH 7.4), the cell pellets were post-fixed in a 1% osmium tetroxide solution in 0.1 M sodium cacodylate buffer (pH 7.4) for 2 h at 4 °C. The bacterial cells were dehydrated in a series of alcohol and acetone and embedded in LR White resin. Ultrathin sections (65 nm) were cut with a diamond knife on a microtome RMC MT-XL (Boeckeler Instruments, Tucson, AZ, USA) collected on copper grids and contrasted with the use of uranyl acetate and Reynold’s liquid. The samples were observed under a LEO-Zeiss 912 AB electron microscope (Carl Zeiss Microscopy, Oberkohen, Germany).

After incubation with the polypeptides, the bacterial pellets were flooded with the first fixative (the same as above described). The next steps of the procedure were performed as above described.

### 4.8. Other Methods

The concentration of protein in the hemolymph was determined using the Bradford method and bovine serum albumin as a standard [47]. The quantity of apoLp-III was determined by weight, while the concentration of defensin was measured by amino acid analysis [48].

### 4.9. Statistical Analysis

Each experiment was performed at least three times. The experimental values are given as means ± SD. The statistical significance of the differences between the control and test values was evaluated using Student’s *t*-test.

## 5. Conclusions

In conclusion, (1) *G. mellonella* defense factors, defensin and apoLp-III exhibited anti-*L. dumoffii* activity, (2) *L. dumoffii* growing on choline-supplemented medium were more sensitive to defensin and apoLp-III, (3) the destructive effect of defense factors studied on bacterial cell morphology was evident in TEM images, and (4) our results indicate that insects, such as *G. mellonella*, with their great diversity of antimicrobial factors of different biochemical properties can serve as a rich source of compounds for testing of *Legionella* susceptibility to the defense-related peptides and proteins.
